# Temporal trends in sex differences in dementia care—results from the Swedish registry for cognitive/dementia disorders, SveDem

**DOI:** 10.1038/s41598-025-03055-y

**Published:** 2025-05-29

**Authors:** Tamar Abzhandadze, Minh Tuan Hoang, Pauline Raaschou, Jakob Norgren, Minjia Mo, Christian Molnar, Hong Xu, Laura Kananen, Madeleine Åkerman, Dorota Religa, Maria Eriksdotter, Sara Garcia-Ptacek

**Affiliations:** 1 Division of Clinical Geriatrics, Department of Neurobiology, Care Sciences and Society, Karolinska Institutet, NEO, Blickagången 16, 141 52 Stockholm, Sweden; 2 Department of Occupational Therapy and Physiotherapy, Sahlgrenska University Hospital, Bruna Stråket 11B, 41346 Gothenburg, Sweden; 3 Department of Medical Epidemiology and Biostatistics, Karolinska Institutet, NEO Blickagången 16, 141 52 Stockholm, Sweden; 4 Faculty of Social Sciences (Health Sciences) and Gerontology Research Center, Tampere University, Kalevantie 4, 33100 Tampere, Finland; 5 Clinical Pharmacology Unit, Clinical Epidemiology Division, Department of Medicine, Karolinska Institutet, 171 77 Stockholm, Sweden; 6 Department of Neurobiology, Care Sciences and Society, Karolinska Institutet, NEO Blickagången 16, 141 52 Stockholm, Sweden; 7 Theme Inflammation and Aging, Karolinska University Hospital, Hälsovägen 13, 141 57 Huddinge, Sweden

**Keywords:** Gender difference, Diagnostic, Equity, Epidemiology, Healthcare, Health care, Neurology

## Abstract

The Swedish Registry for Cognitive/Dementia Disorders (SveDem) follows the quality and equity of dementia care across Sweden. In this study, we investigated temporal trends in sex-based differences in the provision of dementia care. Outcomes were diagnostic work-up, assessments performed by health care professionals, medication use, and social support (defined as initiating contact with a social worker and/or support to relatives). Revisions in dementia diagnosis between baseline and follow-up were evaluated as a marker of diagnostic stability. We included 100,534 individuals diagnosed with dementia between 2008 and 2021 (median age 80 years, 58% women). Dementia registrations rose from 2008 to 2014, then declined after 2015. Alzheimer’s dementia was more frequent in women than men (35% versus 17%), while vascular dementia was less frequent (17% versus 21%). Small differences were observed in dementia care outcomes between sexes. Where differences reached statistical significance, effect sizes were minimal. Dementia diagnosis was revised in 5% of men and 4% of women over a median period of 11 months between baseline and first follow-up. Our results revealed negligible temporal trends in sex-based differences in dementia care among individuals included in the SveDem. However, as SveDem covers only about one third of Sweden’s dementia population, findings may not fully represent national trends.

## Introduction

Sex-based differences in dementia have garnered increasing attention in recent years, with women presenting higher rates of Alzheimer’s disease (AD) diagnosis^1^. While longer life expectancy in women partially explains this disparity, it does not account for all observed differences^2,3^. Sex differences in the risk conferred by ApoE^4^, hormonal changes after menopause^1^ and structural and functional brain differences may predispose women to distinct trajectories of cognitive decline^1,2,5^.

Recognizing sex-based differences in the diagnostics and care of dementia is essential for ensuring equitable healthcare^1^. Dementia care, as defined in this study, refers to the set of healthcare practices and support services provided to individuals living with dementia^6,7^. This includes the diagnostic work-up (e.g., cognitive assessments and neuroimaging), assessments conducted by healthcare professionals, prescription of medications, and support services such as engagement of social workers or family member support^6,7^. Previous research has identified significant disparities in the management of dementia care between men and women, including differences in intervention intensities and the use of diagnostic procedures^8,9^; however, these variations appear to be more closely related to age than to sex. In Sweden, individuals who live alone have also been shown to receive their diagnoses later, where a higher proportion of women than men live alone^10^. We also examined changes in diagnoses between baseline and follow-up, we considered this an indirect measure of diagnostic accuracy or stability since in the Swedish Registry for Cognitive/Dementia Disorders (SveDem), revisions or changes in diagnoses are more frequent among patients with unspecified dementia disorders^11^.

Given the observed sex-based differences in age-adjusted dementia incidence that are underpinned by biological factors and potential disparities in care, it is crucial to investigate whether these differences are mirroring differences in diagnostic practices and treatment approaches. The primary aim of this study was to explore temporal trends in sex differences in dementia care among patients registered in SveDem. A secondary aim was to investigate sex-based differences in the revision of dementia diagnosis between baseline and the one-year follow-up.

## Methods

This register-based and explorative study followed the REporting of studies Conducted using Observational Routinely-collected health Data (RECORD) statement^12^.

### Ethics

This register-based study was approved by the Swedish Ethical Review Authority (registration number: 2021-06246-01; amendment number: 2024-03367-02 [date: 2024-06-13]). The Swedish Ethical Review Authority has waived the need for written informed consent for this study. Patients and caregivers were notified of their registration in SveDem and were given the option to opt-out. Under the Personal Data Act (SFS 1998:204), informed consent was not required for retrieving data from quality registers. Researchers received pseudonymized data from the registries, which included record IDs. The key codes for these record IDs were stored securely at the National Board of Health and Welfare and statistics Sweden. The research was performed in accordance with the ethical guidelines and regulations.

### Design and study population

In this nationwide, explorative and register-based cohort study, we retrieved real-world data from Swedish registries. SveDem, a quality registry for cognitive/dementia disorders, collects data at the time of dementia diagnosis and during annual follow-up^13^. Established in 2007, it has become one of the largest clinical dementia registries globally, capturing ~ 29% of Sweden’s dementia diagnoses by 2021^14^. In addition to recording variables such as age, sex, diagnostic work-up, dementia diagnoses, treatment, and support, the registry also collects data aligned with the Swedish national quality indicators for dementia care^15^. We also used data from the Swedish National Patient Register, which includes diagnoses since 2001, to calculate the Charlson Comorbidity Index (CCI)^16^. We obtained civil status and country of birth data from the Longitudinal Integrated Database for Health Insurance and Labor Market^17^. The unique Swedish personal identification numbers facilitated data linkages across these sources.

We included all individuals registered in SveDem who were diagnosed with dementia between January 1, 2008, and December 31, 2021. Dementia diagnoses extracted from the registry included AD, mixed dementia, vascular dementia (VaD), dementia with Lewy bodies (DLB), Parkinson’s disease dementia (PDD), frontotemporal dementia (FTD), unspecified dementia, and other dementia types (Supplementary Table 1). Diagnoses were retrieved at baseline and the first follow-up. Individuals with missing data on diagnosis, or diagnosis of mild cognitive impairment were excluded.

### Variables and definitions

All outcomes were binary. They reflected multiple aspects of dementia care. The outcomes were extracted from SveDem and categorized into four groups (Supplementary Table 2):

- Dementia work-up—included the administration of one or more of the following tests:
  o The Mini-Mental State Examination (MMSE)^18^, the clock drawing test^19^, the Montreal Cognitive Assessment (MoCA)^20^, or the Rowland Universal Dementia Assessment Scale (RUDAS)^21^. RUDAS and MoCA data were only included between 2018 and 2021, as these tests were not routinely administered before 2018.
  o Structural brain imaging was performed via computed tomography scan (CT) and/or magnetic resonance imaging (MRI).
  o Blood tests were part of the standard dementia workup.
  o In some cases, lumbar punctures were performed to analyze biomarkers in cerebrospinal fluid.
- Assessments by an occupational therapist (OT), physiotherapist (PT), speech and language therapist, or neuropsychologist.
- Medications included were cholinesterase inhibitors (donepezil, galantamine, and rivastigmine) or memantine in participants who had been diagnosed with either AD or mixed dementia. The use of antipsychotics was analyzed for all participants.
- Support was defined as the initiation of contact with a social worker and/or the provision of support to relatives.

The CCI for each participant was calculated before their date of dementia diagnosis using a modified algorithm commonly applied in register-based research in Sweden^22^. Dementia diagnosis codes were excluded from the CCI calculations because all patients had a dementia diagnosis.

### Statistical analysis

SveDem registrations per 100,000 inhabitants were calculated based on the number of dementia cases recorded in the database, including diagnoses from both primary and specialist care centers logged in 2008–2021. Population size by sex for each year (as of December 31, 2021) was obtained from Statistics Sweden, and the dementia registration rates were adjusted accordingly.

Binary logistic regression analysis was performed using generalized linear models with a logit link function to investigate sex-based differences in dementia care^23^. All outcomes were binary and corresponded to key components of dementia care, including diagnostic work-up, assessments by healthcare professionals, pharmacological treatment, and support services. Detailed definitions of these outcomes are provided in the “Variables and definitions” section.

One separate regression model was constructed for each outcome. All models included sex, year of diagnosis, age, and quadratic age (age^2^) as covariates to account for potential non-linear effects of age. These variables were selected based on the aims of the study and to account for age, a well-known and strong determinant of dementia progression and care needs. The inclusion of quadratic age allowed the models to capture more complex age-related patterns, recognizing that the relationship between age and diagnostic workup or treatment may not be strictly linear. To evaluate the significance of the interaction effect (sex × diagnosis year), we fitted a reduced model that excluded the interaction terms. A likelihood ratio test was used to compare the full model with the reduced one, assessing whether the interaction between sex and year of diagnosis significantly improved model fit. For all logistic regressions, we reported the proportions of men and women with corresponding 95% confidence intervals, p-values for sex-based differences, and year-wise differences.

In sensitivity analyses, we included the MMSE score and the CCI as additional covariates, as these represent key factors that are commonly used to determine the extent of diagnostic workups. MMSE was excluded from the primary models because it was also one of the outcomes of interest (i.e., whether MMSE was administered), and its inclusion could have introduced over-adjustment. However, to assess whether cognitive status might influence care practices, we explored its role in supplementary models. Similarly, CCI was not included in the primary models but was examined in sensitivity analyses to explore whether comorbidity burden may account for observed differences in dementia care. The models included sex, year of diagnosis, age, and quadratic age (age^2^).

Revisions to dementia diagnosis were analyzed by comparing baseline records with those from the first follow-up (median: 11 months; interquartile range [IQR]: 7 months) using cross-tabulation.

Data were analyzed using RStudio version 4.2.2 (R Project for Statistical Computing, Vienna, Austria), with statistical significance set at 5%.

## Results

### Participant characteristics

**Fig. 1:**
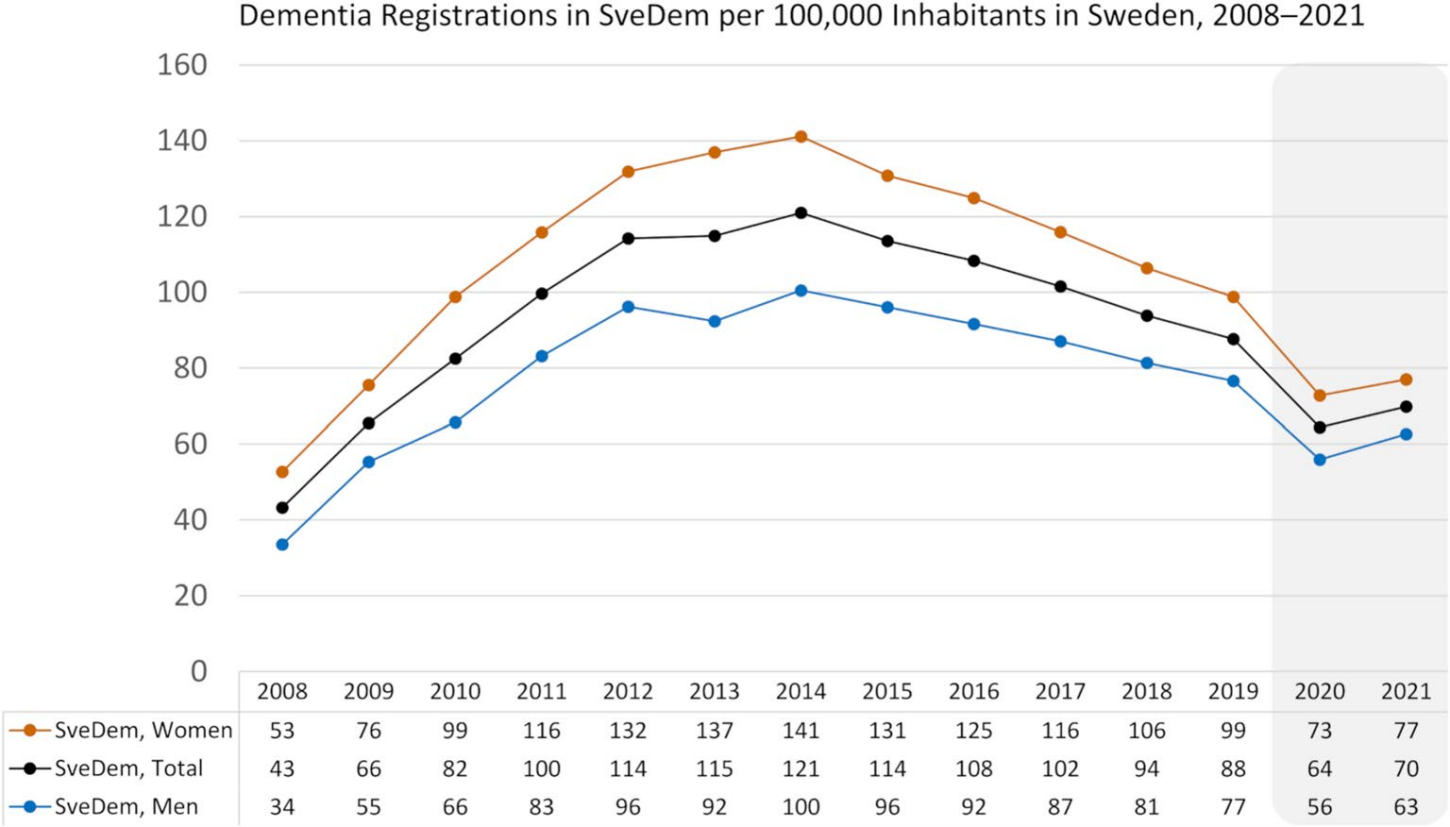
Dementia registrations in the Swedish Registry for Cognitive/Dementia Disorders (SvrDem) per 100,000 inhabitants in Sweden between 2008 and 2021, stratified by men, women, and total registrations. All participants with dementia registrations in SveDem were included. The gray box highlights the COVID-19 pandemic years in Sweden. The results were not standardized by age.

The baseline SveDem sample included 101,071 registrations. After applying the inclusion criteria, 100,534 patients with dementia were retained (Supplementary Fig. 1). Median (IQR) age was 80 (10) years; 79 (10) years for men and 81 (10) years for women, 58% were women (Supplementary Table 3). Among both men and women, the number of dementia registrations in SveDem increased steadily between 2008 and 2014 before declining in 2015 (Fig. 1). Older age groups exhibited higher registration rates (Supplementary Fig. 2). Descriptive information stratified by sex for various aspects of dementia care, as well as the registration ratio, is presented in Table 1.

**Table 1:** Dementia disorders, year of diagnosis, and variables related to diagnostic work-up, medication, and support, stratified by sex (prevalence ratio: > 1 indicates a higher prevalence among women, while < 1 indicates a higher prevalence among men).

	Women n = 58,286	Men n = 42,248	Prevalence Ratio	Overall n = 100,534
	n (%)	n (%)	P_Women_/P_Men_	n (%)
Type of diagnostic unit, yes				
Primary care	27,225 (47)	18,241 (43)	1.09	45,466 (45)
Specialist care	31,061 (53)	24,007 (57)	0.93	55,068 (55)
Type of dementia diagnosis				
Alzheimer’s disease	27,225 (47)	12,001 (28)	1.25	32,647 (32)
Unspecified dementia	31,061 (53)	8568 (20)	1.15	21,737 (22)
VaD	27,225 (47)	9073 (21)	0.81	19,097 (19)
Mixed dementia	31,061 (53)	8109 (19)	1.00	19,067 (19)
Other dementias	27,225 (47)	1293 (3)	0.67	2580 (3)
PDD	31,061 (53)	1006 (2)	0.50	1517 (2)
FTD	27,225 (47)	772 (2)	0.50	1637 (2)
LBD	826 (1)	1426 (3)	0.33	2252 (2)
Calendar year of diagnosis				
2008	1961 (3)	1217 (3)	1.00	3178 (3)
2009	2838 (5)	2028 (5)	1.00	4866 (5)
2010	3753 (6)	2452 (6)	1.00	6205 (6)
2011	4445 (8)	3133 (7)	1.14	7578 (8)
2012	5096 (9)	3658 (9)	1.00	8754 (9)
2013	5326 (9)	3565 (8)	1.13	8891 (9)
2014	5538 (10)	3911 (9)	1.11	9449 (9)
2015	5164 (9)	3769 (9)	1.00	8933 (9)
2016	4976 (9)	3645 (9)	1.00	8621 (9)
2017	4658 (8)	3505 (8)	1.00	8163 (8)
2018	4310 (7)	3313 (8)	0.88	7623 (8)
2019	4045 (7)	3138 (7)	1.00	7183 (7)
2020	2991 (5)	2311 (5)	1.00	5302 (5)
2021	3185 (5)	2603 (6)	0.83	5788 (6)
Diagnostic work-up, yes				
MMSE*	54,510 (96)	39,622 (96)	1.00	94,132 (96)
Clock Test^†^	50,381 (89)	37,097 (90)	0.99	87,478 (89)
RUDAS^‡^	385 (3)	274 (2)	1.50	659 (2)
MoCA^§^	1939 (21)	1677 (22)	0.95	3616 (22)
MRI-CT^||^	50,901 (90)	38,135 (92)	0.98	89,036 (91)
Blood analysis^{^	54,521 (96)	39,586 (96)	1.00	94,107 (96)
Lumbar puncture^#^	14,388 (26)	11,784 (29)	0.90	26,172 (27)
Assessments, yes				
OT/PT**	26,572 (47)	18,914 (46)	1.02	45,486 (46)
Speech therapist^††^	1520 (3)	1205 (3)	1.00	2725 (3)
Neuropsychologist^‡‡^	8856 (16)	7839 (19)	0.84	16,695 (17)
Medications, yes				
Cholinesterase inhibitors^§§^	18,804 (60)	11,850 (60)	1.00	30,654 (60)
Memantine^||||^	4956 (16)	3809 (19)	0.84	8765 (17)
Antipsychotics^{{^	3333 (6)	2279 (6)	1.00	5612 (6)
Support, yes				
Social worker^##^	6541 (12)	5670 (14)	0.86	12,211 (13)
Support to relatives***	34,892 (65)	26,334 (67)	0.97	61,226 (66)

PDD, Parkinson’s Disease Dementia; FTD, Frontotemporal Dementia; LBD, Lewy Body Dementia; VaD, Vascular Dementia; MMSE, Mini-Mental State Examination; RUDAS, Rowland Universal Dementia Assessment Scale; MoCA, Montreal Cognitive Assessment; MRI-CT, Magnetic Resonance Imaging-Computed Tomography; OT, occupational therapist; PT, physiotherapist. MoCA and RUDAS are alternative options for MMSE. Memantine and Cholinesterase inhibitors: calculated on a subgroup of patients with Alzheimer’s dementia and Mixed dementia. Regression analysis for RUDAS and MoCA, period 2018–2021. Missing values n (%): *2412 (2.4), ^†^2412 (2.4), ^‡^74,495 (74.1), ^§^84,046 (83.6), ^II^2312 (2.3), ^{^ 2412 (2.4), ^#^3418 (3.4), **3518 (3.5), ^††^3719 (3.7), ^‡‡^4222 (4.2), ^§§^1508 (1.5), ^IIII^2211(2.2), ^{{^5529 (5.5), ^##^44,926 (4.9), *** 7338 (7.3).

**Fig. 2:**
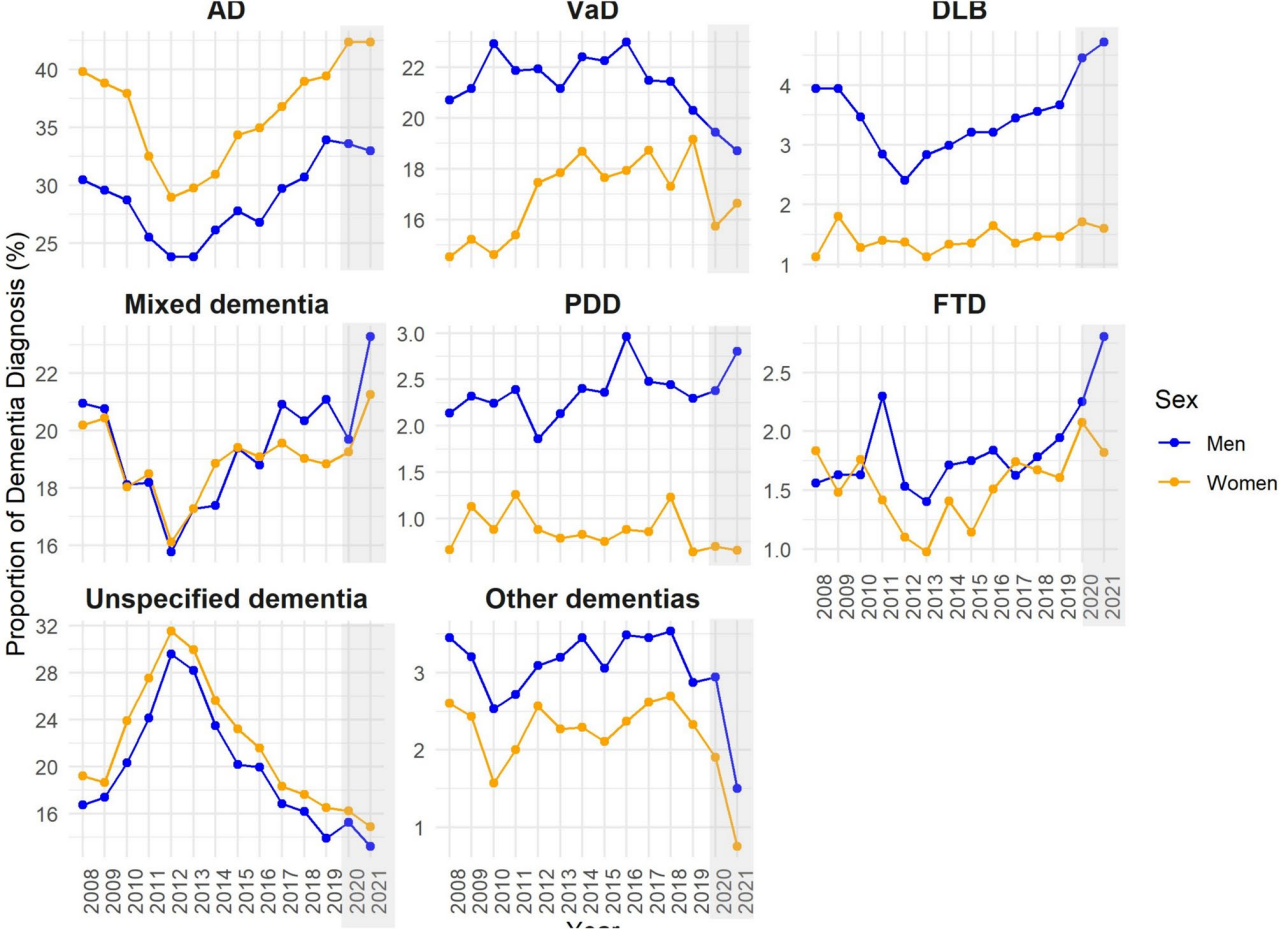
Proportion of dementia diagnosis in the Swedish Registry for Cognitive/Dementia Disorders by type, sex, and year (2008–2021). The gray box highlights the COVID-19 pandemic years in Sweden. AD, Alzheimer’s disease; VaD, vascular dementia; DLB, dementia with Lewy bodies; PDD, Parkinson’s disease dementia; FTD, frontotemporal dementia.

AD diagnosis was 25% more common among women than men, whereas VaD diagnosis was 19% more common in men than women, Table 1. Women had a relatively higher proportion of Alzheimer’s disease (AD) diagnoses (Fig. 2), particularly in the older age groups (75–84 and ≥ 85 years), while men more frequently received diagnoses of vascular dementia (VaD), dementia with Lewy bodies (DLB), Parkinson’s disease dementia (PDD), and other dementia types (Fig. 2, Supplementary Fig. 3). DLB and PDD were especially common among men aged 65–74 and 75–84. Additionally, unspecified and mixed dementia were more common among women in the oldest age group (≥ 85 years) (Supplementary Fig. 3).

### Sex-based differences in dementia care

Multivariable binary logistic regression models, adjusted for the year of diagnosis, age, and quadratic age, revealed statistically significant sex-based differences across outcomes and changes in these differences over time (Table 2, Supplementary Figs. 4–7). However, the magnitude of these differences was generally small. The largest difference was found for performance of lumbar puncture (men 28.84% CI 28.66–29.01 versus women 25.56; CI 25.41–25.70). Between 2008 and 2021, statistically significant, but small changes were seen in the administration of MMSE, MRI/CT use, memantine and cholinesterase inhibitor prescriptions, and the initiation of contact with a social worker and/or the provision of support to relatives (Table 2). Sensitivity analyses adjusted for baseline MMSE score revealed similar patterns (Supplementary Table 4). Additional adjustments for CCI did not substantially change the overall pattern of sex-based differences in diagnosis and treatment (Supplementary Table 5). Both sensitivity analyses were adjusted for age.

**Table 2:** Sex-based differences in dementia care (2008–2021). The regression models included sex as a primary explanatory variable, along with diagnosis year and age at the time of dementia diagnosis.

Outcome	Sex	Estimated proportion (95% CI), 2008–2021	P value, sex-based differences	P value, sex-based differences over time
Diagnostic work-up				
MMSE	Men	95.98 (95.96–95.99)	0.97	< 0.05
	Women	95.98 (95.97–96.00)		
Clock Test	Men	89.75 (89.70–89.81)	< 0.001	0.25
	Women	88.65 (88.60–88.70)		
RUDAS^#^	Men	2.40 (2.39–2.42)	0.24	0.71
	Women	2.64 (2.63–2.66)		
MoCA^#^	Men	22.56 (22.47–22.66)	0.09	0.89
	Women	21.47 (21.39–21.55)		
MRI-CT	Men	92.16 (92.10–92.22)	< 0.001	0.02
	Women	89.60 (89.53–89.65)		
Blood analysis	Men	95.91 (95.89–95.92)	0.72	0.87
	Women	95.96 (95.94–95.97)		
Lumbar puncture	Men	28.84 (28.66–29.01)	< 0.001	0.45
	Women	25.56 (25.41–25.70)		
Assessments				
OT/PT	Men	46.32 (46.25–46.39)	< 0.01	0.36
	Women	47.27 (47.21–47.33)		
Speech therapist	Men	2.96 (2.94–2.98)	0.02	0.36
	Women	2.71 (2.69–2.73)		
Neuropsychologist	Men	19.33 (19.23–19.43)	< 0.001	0.35
	Women	15.88 (15.80–15.95)		
Medications				
Cholinesterase inhibitors	Men	59.80 (59.63–59.99)	0.20	0.02
	Women	60.38 (60.23–60.53)		
Memantine	Men	19.37 (19.31–19.44)	< 0.001	0.01
	Women	16.04 (15.99–16.09)		
Antipsychotics	Men	5.59 (5.57–5.61)	0.02	0.69
	Women	5.96 (5.95–5.97)		
Support				
Social worker	Men	14.05 (13.97–14.14)	< 0.001	0.03
	Women	11.83 (11.76–11.90)		
Support to relatives	Men	66.87 (66.78–66.97)	< 0.001	0.04
	Women	64.82 (64.73–64.91)		

^*#*^Only for 2018–2021, owing to variable introduction in later years. Statistics: binary logistic regression.

MMSE, Mini-Mental State Examination; RUDAS, Rowland Universal Dementia Assessment Scale; MoCA, Montreal Cognitive Assessment; MRI-CT, Magnetic Resonance Imaging-Computed Tomography; OT, occupational therapist; PT, physiotherapist. Memantine and Cholinesterase inhibitors: calculated on a subgroup of patients with Alzheimer’s dementia and Mixed dementia. Regression analysis for RUDAS and MoCA performed between 2018 and 2021.

Binary logistic regression model example: Regression model <—glm(Memantine ~ Sex * Diagnose_year + Age + Age2, data = dat_df, family = binomial(link = “logit”)).

### Sex-based differences in changes to dementia diagnosis from baseline to first follow-up

A total of 48,433 patients had available baseline and first follow-up data, with a median interval of 11 months (IQR: 7 months) between assessments. Among these, 20,568 (43%) were men and 27,865 (57%) were women. Dementia diagnosis was revised in 5% of men (Table 3, Panel A) and 4% of women (Table 3, Panel B), resulting in an overall diagnostic revision rate of 4.5% (Supplementary Table 6).

**Table 3:** Changes in dementia diagnoses from baseline to first follow-up among men (n ═ 20,568, Panel A) and women (n = 27,865, Panel B).

Panel A	(n)	Changes in dementia diagnosis from baseline to first follow-up among men (total revision rate, 5%)								
		Mixed dementia	Unspecified dementia	AD	PDD	FTD	DLB	Other dementias	VaD	MCI
Baseline dementia diagnoses	Mixed dementia (3,778)	**3684 (97.5)**	15 (0.4)	32 (0.8)	0	1 (0)	15 (0.4)	2 (0.1)	21 (0.6)	8 (0.2)
	Unspecified dementia (4,234)	96 (2.3)	**3722 (87.9)**	190 (4.5)	11 (0.3)	18 (0.4)	47 (1.1)	20 (0.5)	112 (2.6)	18 (0.4)
	AD (7,112)	49 (0.7)	32 (0.4)	**6968 (98)**	5 (0.1)	6 (0.1)	21 (0.3)	3 (0.0)	11 (0.2)	17 (0.2)
	PDD (448)	1 (0.2)	1 (0.2)	2 (0.4)	**436 (97.3)**	1 (0.2)	6 (1.3)	0 (0)	1 (0.2)	0 (0)
	FTD (413)	3 (0.7)	3 (0.7)	5 (1.2)	0 (0)	**396 (95.9)**	2 (0.5)	1 (0.2)	0 (0)	3 (0.7)
	DLB (817)	3 (0.4)	6 (0.7)	6 (0.7)	1 (0.1)	2 (0.2)	**791 (96.8)**	3 (0.4)	3 (0.4)	2 (0.2)
	Other dementias (560)	6 (1.1)	15 (2.7)	19 (3.4)	1 (0.2)	2 (0.4)	7 (1.3)	**492 (87.9)**	15 (2.7)	3 (0.5)
	VaD (3,206)	61 (1.9)	13 (0.4)	17 (0.5)	3 (0.1)	0 (0)	6 (0.2)	5 (0.2)	**3090 (96.4)**	11 (0.3)

Panel B		Change in dementia diagnosis from baseline to first follow-up among women (total revision rate, 4%)								
Baseline dementia diagnoses	Mixed dementia (4,760)	**4686 (98.4)**	15 (0.3)	30 (0.6)	2 (0.0)	4 (0.1)	5 (0.1)	2 (0)	12 (0.3)	4 (0.1)
	Unspecified dementia (6,373)	120 (1.9)	**5661 (88.8)**	345 (5.4)	4 (0.1)	25 (0.4)	21 (0.3)	20 (0.3)	162 (2.5)	15 (0.2)
	AD (11,612)	68 (0.6)	35 (0.3)	**11,432 (98.4)**	2 (0.0)	8 (0.1)	15 (0.1)	11 (0.1)	13 (0.1)	28 (0.2)
	PDD (224)	0 (0)	4 (1.8)	1 (0.4)	**217 (96.9)**	0 (0)	0 (0)	0 (0)	2 (0.9)	0 (0)
	FTD (415)	1 (0.2)	1 (0.2)	6 (1.4)	0 (0)	**401 (96.6)**	0 (0)	4 (1.0)	1 (0.2)	1 (0.2)
	DLB (400)	2 (0.5)	5 (1.3)	1 (0.3)	1 (0.3)	2 (0.5)	**388 (97.0)**	0 (0)	1 (0.3)	0 (0)
	Other dementias (582)	9 (1.5)	13 (2.2)	17 (2.9)	0 (0)	5 (0.9)	3 (0.5)	**519 (89.2)**	14 (2.4)	2 (0.3)
	VaD (3,499)	64 (1.8)	21 (0.6)	26 (0.7)	0 (0)	2 (0.1)	4 (0.1)	4 (0.1)	**3369 (96.3)**	9 (0.3)

Bold text in diagonal cells indicate n (%) of participants that retained the same diagnosis from baseline to first follow-up (median 11 months).

AD, Alzheimer’s dementia; PDD, Parkinson’s Disease Dementia; FTD, Frontotemporal Dementia; LBD, Lewy Body Dementia; VaD, Vascular Dementia; MCI, Mild Cognitive Impairment.

## Discussion

This nationwide cohort study in Sweden demonstrated a steady increase in dementia registrations between 2008 and 2014, followed by a decline after 2015. Women consistently exhibited higher registration rates than men, which may be attributed to several factors, including an overall higher incidence of dementia. Women had a higher proportion of AD, while men had higher proportions of VaD, DLB, PDD, and other dementia types. Comparisons between men and women revealed minimal differences in revised diagnosis. Slight variations were also observed regarding the use of dementia work up, assessments by health care professionals, medications, and support measures.

There may be several explanations for the overall decline in SveDem dementia registrations since 2015. First, it may be related to changes within the registry itself. The earlier increase in registrations was partially driven by a governmental initiative aimed at boosting primary care registrations through financial incentives between 2012 and 2014, followed by a return to pre-initiative levels once the program ended. It may also reflect reduced healthcare capacity^24^. Another sharp decline was observed between 2019 and 2020; however, this decline could be attributed to the coronavirus disease 2019 (COVID-19) pandemic, which led to increased mortality among vulnerable individuals and closed memory clinics. We recently reported a decline in the average number of new dementia registrations across the pre-COVID-19, COVID-19, and post-COVID-19 periods^25^. The average nationwide monthly registrations decreased from 595 cases pre-COVID-19 to 415 cases during COVID-19, with a slight increase to 470 cases afterward. This downward trend was observed in both primary care and specialized memory clinics and persisted into the post-COVID-19 period^25^.

A true decline in the incidence of dementia between 2008 to 2021 may have occurred, as is supported by findings from several studies conducted in Europe and North America^26,27^. Those studies attributed the decrease in dementia incidence to improvements in cardiovascular health, education, and cognitive reserves over recent decades^26–28^. Better management of cardiovascular risk factors such as hypertension, as well as lifestyle improvements that include high levels of physical activity, have also been suggested to be major contributors^26,29^. Higher educational attainment and improved psychosocial working conditions over time may also have enhanced cognitive reserves, contributing to the observed decrease in dementia incidence^26,27,30^. However, the limited coverage of the SveDem registry makes it difficult to draw conclusions about the underlying dementia incidence in Sweden.

The decline in dementia registrations was similar across the sexes; however, the gap between women and men was greater in high-registration years (2013–2016). Although the gap narrowed slightly during the pandemic years, women remained more frequently registered in SveDem across all years. This difference may be attributed to the complex interplay of biological, social, and environmental factors^2,10,31–33^. The diagnostic rates of various dementia types remained stable over the study period, with notable sex-based differences in AD and VaD. The proportion of patients with AD was consistently higher for women than men, likely because of their longer average life expectancy. In contrast, VaD was slightly more common in men than women but remained generally stable over time. This may be because men have a higher incidence of stroke than women, particularly between the ages of 45 and 75 years^34^, which may increase their likelihood of developing vascular dementia later. A trend was observed in the diagnosis of unspecified dementia, with a noticeable increase beginning in 2012 followed by a decline starting in 2015. This pattern may be attributable to a governmental initiative implemented between 2012 and 2014 that used financial incentives to encourage primary care registrations.

Revisions to dementia diagnosis between baseline and the first follow-up were minimal. Both men and women most often experienced a shift from unspecified dementia at baseline to a diagnosis of AD at the first follow-up. This may be attributable to the diagnostic procedure for dementia. It is possible that initially, the patient received a “working” diagnosis in order to access social services or care while work-up was being conducted. For example, if patients were referred to specialist care or if more specialized tests (such as genetics, or PET) were required. As testing progressed, more information was gathered, discussions with relatives took place, and the diagnosis could be refined and specified. Another explanation for this shift may be that disease progression could lead to more pronounced and distinguishable clinical features over time, facilitating more definitive diagnoses^35^.

Our results partially align with those of Religa et al.^8^ in the early years of the SveDem registry, who first reported that men underwent more diagnostic tests, such as lumbar punctures and MRIs, than women. However, after adjusting for age, these differences disappeared, indicating that the disparities were age-related rather than sex-specific, with older women generally undergoing fewer tests^8^. Koikkalainen et al.^36^ highlighted the growing importance of imaging data in the differential diagnosis of neurodegenerative diseases and the positive trend in MRI/CT usage can be interpreted as a marker of improved quality of dementia care. The observed differences in medication usage, particularly concerning the higher prescription rate of memantine in men than women, align with previous studies that reported sex-specific patterns regarding dementia treatment^37^. Although no differences have been reported between men and women concerning the cognitive and functional effectiveness of memantine^38^, the drug may be more commonly prescribed to men to better manage certain behavioral symptoms of AD, such as agitation^1,39^. The higher prevalence of cardiovascular disease in men than women may also influence the safety and prescribing patterns of cholinesterase inhibitors, which can have cardiac side effects^40^. Despite these concerns, several studies have demonstrated the beneficial outcomes associated with cholinesterase inhibitor use, including slower cognitive decline^41^, reduced stroke risk^42^, and lower all-cause mortality among patients with AD who have histories of myocardial infarction^43^. Our findings reinforce the complexity of sex and age influences on both diagnostic and therapeutic approaches in dementia care.

We found that caregivers of male participants diagnosed with dementia were more likely to receive support than those caring for female ones. This disparity may stem from societal and cultural factors that shape caregiving roles and support systems^44^. Moreover, we found significant temporal trends in family support which increased over time. The observed disparities in family support emphasize the need for tailored interventions to address the unique needs of individuals with dementia and their families throughout the diagnostic and treatment processes^45^.

This study had several strengths and limitations. One primary limitation is the potential for selection bias; SveDem covers ~ 30% of the expected dementia cases in Sweden^11^, and may not fully represent the national population with the disease. This limited coverage may have resulted in findings that do not accurately reflect the broader demographic and clinical characteristics of all patients with dementia in Sweden. This reduced coverage also makes it impossible to draw conclusions about the real incidence of dementia in Sweden or its changes over time. A second limitation is the retrospective nature of the analysis, which was inherent to the dataset used. A significant strength of this study is the large size and comprehensiveness of the SveDem registry, the largest clinical quality dementia registry of its kind. Nevertheless, the SveDem registry provides real-world data that reflect clinical practices in the diagnosis, treatment, and care of dementia, enhancing the likelihood of accurately capturing dementia management across both primary and specialized care facilities. The dataset spans 14 years, offering a comprehensive view of long-term trends in care practices between 2008 and 2021. A third limitation concerns the generalizability of our findings. Regional variations in registry participation and access to dementia care could limit the generalizability of the findings, as certain subgroups—such as individuals in rural areas or with lower socioeconomic status—may be underrepresented. This limited coverage may result in findings that do not fully capture the broader demographic and clinical characteristics of all individuals with dementia in Sweden. Moreover, Sweden’s tax-financed healthcare system ensures equitable access to healthcare services, suggesting that our findings may only apply to countries with similar healthcare systems.

In conclusion, our results revealed negligible temporal trends in sex-based differences in dementia care among individuals included in the SveDem. These results may indicate that Sweden’s gender-equality initiatives in healthcare have largely met their objective of delivering care that is both equitable and consistent. However, as SveDem covers only about one third of Sweden’s dementia population, findings may not fully represent national trends.

## Supplementary Information

Supplementary Information.

## Data Availability

The datasets generated and analyzed during the current study are not publicly available due to Swedish and EU legislation as the datasets include sensitive health data. However, federated analyses are possible by contacting the senior researcher (sara.garcia-ptacek@ki.se).
