# Supplementary material for: Temporal trends in sex differences in dementia care—results from the Swedish registry for cognitive/dementia disorders, SveDem
Source: Sci Rep. 2025 May 29;15:18890. doi: 10.1038/s41598-025-03055-y (PMC12122826; doi:10.1038/s41598-025-03055-y)
Supplement: Supplementary file 1 — Supplementary Information. [file 41598_2025_3055_MOESM1_ESM.docx]

# SUPPLEMENTARY MATERIAL

### Temporal trends in sex differences in Dementia Care - results from the Swedish registry for cognitive/dementia disorders, SveDem.

**Supplementary Table 1.** Dementia diagnosis codes in the Swedish Quality Registry for Cognitive/Dementia Disorders (SveDem)

| ICD-10 codes in SveDem | Dementia diagnosis | Categorizations used in the study |
| --- | --- | --- |
| F00.0 | Dementia in Alzheimer's disease with early onset | Alzheimer's disease |
| F00.1 | Dementia in Alzheimer's disease with late onset | Alzheimer's disease |
| F00.2 | Dementia in Alzheimer's disease, atypical or mixed type | Mixed dementia |
| F01.0 | Vascular dementia with acute onset | Vascular dementia |
| F01.1 | Multi-infarct dementia | Vascular dementia |
| F01.2 | Subcortical vascular dementia | Vascular dementia |
| F01.3 | Mixed cortical and subcortical vascular dementia | Vascular dementia |
| F01.8 | Another specified vascular dementia | Vascular dementia |
| F01.9 | Unspecified vascular dementia | Vascular dementia |
| F02.0 | Frontotemporal dementia | Frontotemporal dementia |
| F02.1 | Dementia in Creutzfeldt-Jakob disease | Other dementias |
| F02.2 | Dementia in Huntington's disease | Other dementias |
| F02.3 | Dementia in Parkinson's disease | Parkinson's disease dementia |
| F02.4 | Dementia in disease caused by human immunodeficiency virus | Other dementias |
| F02.8 | Lewy body dementia | Lewy Body Dementia |
| F03.9 | Unspecified dementia | Unspecified dementia |
| F10.7A | Alcohol-related dementia | Other dementias |
| Abbreviation: ICD-10: 10th revision of the International Statistical Classification of Diseases and Related Health Problems. | | |

**Supplementary Table 2.** Dementia care outcomes in the study.

| **Outcomes of this study** |
| --- |
| Diagnostic work-up |
| - Cognitive screenings with the Mini-Mental State Examination (MMSE), clock test, the Montreal Cognitive Assessment (MoCA), or the Rowland Universal Dementia Assessment Scale (RUDAS). The RUDAS can be used for those with dementia whose mother tongue is not Swedish, have different cultural backgrounds, or have lower educational levels. The MoCA can be an alternative to the MMSE when investigating early symptoms,. - Structural brain imaging with computed tomography or magnetic resonance imaging - Blood tests - Lumbar puncture |
| Assessments |
| - Assessments by an occupation therapist - Assessment by a physiotherapist - Assessment by a speech and language therapist - Assessment by a neuropsychologist |
| Medications |
| - Cholinesterase inhibitors (donepezil, galantamine, and rivastigmine), registered in SveDem - Memantine, registered in SveDem |
| - Treatment with antipsychotic medication, registered in SveDem |
| Support |
| - Social worker - the initiation of contact with a social worker. - Support to relatives - the provision of support to relatives |
| Abbreviations: SveDem, the Swedish quality registry for cognitive/dementia disorders. |

**Supplementary Table 3**. Characteristics of the study sample, overall as well as stratified by sex (2008–2021).

| Category | Men  n = 42,248 | | Women  n = 58,286 | | Overall  n = 100,534 | |  |
| --- | --- | --- | --- | --- | --- | --- | --- |
| Age, range 27–105 years |  | |  | |  | |  |
| Mean (SD) | 78.6 (7.8) | | 80.3 (7.9) | | 79.5 (7.9) | |  |
| Median (IQR) | 79 (10) | | 81 (10) | | 80 (10) | |  |
| Mini Mental State Examination score * |  | |  | |  | |  |
| Mean (SD) | 21.2 (5.0) | | 20.7 (4.9) | | 20.9 (4.9) | |  |
| Median (IQR) | 22 (7) | | 21 (6) | | 22 (7) | |  |
| Country of birth, n (%) ^†^ |  | |  | |  | |  |
| Sweden | 37,212 (88.1) | | 50,356 (86.4) | | 87,568 (88.1) | |  |
| Other countries | 5,035 (11.9) | | 7,927 (13.6) | | 12,962 (11.9) | |  |
| Living arrangements, n (%) ^‡^ |  | |  | |  | |  |
| Own accommodation | 38,559 (91.5) | | 52,531 (90.4) | | 91,090 (91.5) | |  |
| Nursing homes | 3,569 (8.5) | | 5,603 (9.6) | | 9,172 (8.5) | |  |
| Cohabitation status, n (%) ^§^ |  | |  | |  | |  |
| Living alone | 11,782 (29.6) | | 32,013 (59.4) | | 43,795 (29.6) | |  |
| Cohabiting | 28,079 (70.4) | | 21,918 (40.6) | | 49,997 (70.4) | |  |
| Communal home care services, n (%) ^II^ | |  | |  | |  | |
| Yes | 10,256 (25.0) | | 21,308 (37.6) | | 31,564 (25.0) | |  |
| No | 30,779 (75.0) | | 35,405 (62.4) | | 66,184 (75.0) | |  |
| Charlson Comorbidity index (CCI), range 0–21 | | |  | |  | |  |
| Mean (± SD) | 1.7 (2.2) | | 1.4 (2.0) | | 1.5 (2.1) | |  |
| Median (IQR) | 1 (3) | | 0 (2) | | 1 (2) | |  |
| CCI, n (%) |  | |  | |  | |  |
| 0 | 16,865 (39.9) | | 29,607 (50.8) | | 46,472 (46.2) | |  |
| 1 | 6,872 (16.3) | | 9,833 (16.9) | | 16,705 (16.6) | |  |
| 2 | 7,919 (18.7) | | 8,486 (14.6) | | 16,405 (10.3) | |  |
| ≥ 3 | 10,592 (25.0) | | 10,360 (17.8) | | 20,952 (20.8) | |  |
| Abbreviations: SD, standard deviation; IQR, interquartile range.  Missing values, n (%): ^*^6,034 (6.4); †4 (< 0.001); ^‡^272 (0.3); ^§^6,742 (6.7); ^II^2,786 (2.8). | | | | | | | |

**Supplementary Table 4.** Sex-based differences in dementia care (2008–2021). The models were adjusted for baseline Mini-Mental State Examination score.

| Outcomes | Sex | Estimated proportion (95% CI), 2008–2021 | P value, sex differences | P value, sex differences over time |
| --- | --- | --- | --- | --- |
| Diagnostic work-up | |  |  |  |
| Clock Test | Men | 92.8 (92.7–92.8) | < 0.001 | 0.06 |
|  | Women | 92.0 (92.0–92.1) |  |  |
| RUDAS^#^ | Men | 1.79 (1.77–1.81) | 0.97 | 0.88 |
|  | Women | 1.78 (1.76–1.80) |  |  |
| MoCA^#^ | Men | 21.1 (20.9–21.2) | 0.23 | 0.86 |
|  | Women | 20.3 (20.2–20.4) |  |  |
| MRI-CT | Men | 92.9 (92.8–92.9) | < 0.001 | 0.05 |
|  | Women | 90.5 (90.5–90.6) |  |  |
| Blood analysis | Men | 96.4 (96.4–96.4) | 0.32 | 0.66 |
|  | Women | 96.5 (96.5–96.5) |  |  |
| Lumbar puncture | Men | 29.4 (29.2–29.6) | < 0.001 | 0.33 |
|  | Women | 26.1 (26.0–26.3) |  |  |
| Assessment | |  |  |  |
| OT/PT | Men | 46.8 (46.8–46.9) | < 0.001 | 0.24 |
|  | Women | 48.0 (48.0–48.1) |  |  |
| Speech therapist | Men | 2.80 (2.78–2.82) | 0.05 | 0.50 |
|  | Women | 2.80 (2.78–2.82) |  |  |
| Neuropsychologist | Men | 19.8 (19.6–20.0) | < 0.001 | 0.81 |
|  | Women | 16.3 (16.2–16.4) |  |  |
| Medications | |  |  |  |
| Cholinesterase inhibitors | Men | 60.0 (59.8–60.2) | 0.05 | 0.01 |
|  | Women | 60.9 (60.8–61.1) |  |  |
| Memantine | Men | 19.3 (19.2–19.4) | < 0.001 | < 0.01 |
|  | Women | 15.8 (15.7–15.9) |  |  |
| Antipsychotics | Men | 5.06 (5.04–5.09) | 0.03 | 0.77 |
|  | Women | 5.40 (5.38–5.42) |  |  |
| Support | | |  |  |
| Social worker | Men | 14.1 (14.0–14.1) | < 0.001 | 0.03 |
|  | Women | 11.9 (11.8–12.0) |  |  |
| Support to relatives | Men | 67.3 (67.2–67.5) | < 0.001 | 0.05 |
|  | Women | 65.4 (65.3–65.5) |  |  |
| *^#^*Note: Only for 2018–2021, owing to limited data availability for previous years. ***Statistics:*** binary logistic regression. Memantine and cholesterol-lowering: calculated on a subgroup of patients with Alzheimer's dementia and mixed dementia. Regression analysis for RUDAS and MoCA, period 2018–2021.  Binary logistic regression model: Regression model <- glm(Outcome ~ Sex* Diagnose_year + Age + Age2 + Baseline_MMSE, data = data_df, family = binomial(link = "logit"))  Abbreviations: MMSE, Mini-Mental State Examination; RUDAS, Rowland Universal Dementia Assessment Scale; MoCA, Montreal Cognitive Assessment; MRI-CT, Magnetic Resonance Imaging - Computed Tomography; OT, occupational therapist; PT, physiotherapist. | | | | |
|  | | | | |

**Supplementary Table 5.** Sex-based differences in dementia care (2008–2021). The models were adjusted for baseline Mini-Mental State Examination score and Charlson comorbidity index.

| Outcomes | Sex | Estimated proportion (95% CI), 2008–2021 | P value, sex differences | P value, sex differences over time |
| --- | --- | --- | --- | --- |
| Diagnostic work-up | |  |  |  |
| Clock Test | Men | 92.8 (92.7–92.8) | < 0.001 | 0.06 |
|  | Women | 92.0 (92.0–92.1) |  |  |
| RUDAS^#^ | Men | 1.79 1.77 1.82 | 0.98 | 0.88 |
|  | Women | 1.78 1.76 1.80 |  |  |
| MoCA^#^ | Men | 21.1 20.9 21.2 | 0.23 | 0.88 |
|  | Women | 20.3 20.2 20.4 |  |  |
| MRI-CT | Men | 92.9 92.8 92.9 | < 0.001 | 0.05 |
|  | Women | 90.5 90.5 90.6 |  |  |
| Blood analysis | Men | 96.4 96.4 96.4 | 0.32 | 0.65 |
|  | Women | 96.5 96.5 96.5 |  |  |
| Lumbar puncture | Men | 29.4 29.2 29.5 | < 0.001 | 0.29 |
|  | Women | 26.1 26.0 26.3 |  |  |
| Assessments | | |  |  |
| OT/PT | Men | 46.8 46.8 46.9 | < 0.001 | 0.24 |
|  | Women | 48.0 48.0 48.1 |  |  |
| Speech therapist | Men | 2.80 2.78 2.83 | 0.05 | 0.50 |
|  | Women | 2.59 2.58 2.61 |  |  |
| Neuropsychologist | Men | 19.8 19.6 20.0 | < 0.001 | 0.81 |
|  | Women | 16.3 16.2 16.4 |  |  |
| Medications | |  |  |  |
| Cholinesterase inhibitors | Men | 60.0 59.8 60.3 | 0.05 | 0.01 |
|  | Women | 60.9 60.8 61.1 |  |  |
| Memantine | Men | 19.3 19.2 19.4 | < 0.001 | < 0.01 |
|  | Women | 15.8 15.8 15.9 |  |  |
| Antipsychotics | Men | 5.06 5.04 5.09 | 0.03 | 0.76 |
|  | Women | 5.40 5.38 5.42 |  |  |
| Support | | |  |  |
| Social worker | Men | 14.1 (14.0–14.1) | < 0.001 | 0.03 |
|  | Women | 11.9 (11.8–12.0) |  |  |
| Support to relatives | Men | 67.3 (67.3–67.4) | < 0.001 | 0.05 |
|  | Women | 65.4 (65.3–65.5) |  |  |
| *^#^*Note: Only for 2018–2021, owing to limited data availability for previous years. ***Statistics:*** binary logistic regression. Memantine and cholesterol-lowering: calculated on a subgroup of patients with Alzheimer's dementia and mixed dementia. Regression analysis for RUDAS and MoCA, period 2018–2021.  Binary logistic regression model: Regression model <- glm(Outcome ~ Sex* Diagnose_year + Age + Age2 + Baseline_MMSE, data = data_df, family = binomial(link = "logit"))  Abbreviations: MMSE, Mini-Mental State Examination; RUDAS, Rowland Universal Dementia Assessment Scale; MoCA, Montreal Cognitive Assessment; MRI-CT, Magnetic Resonance Imaging - Computed Tomography; OT, occupational therapist; PT, physiotherapist. | | | | |

**Supplementary Table 6.** Revision in dementia diagnoses from baseline to first follow-up (median, 11 months; n = 48,433; total revision rate, 4.5%).

| (n) | | Revision in dementia diagnosis from baseline to first follow-up, n (%) | | | | | | | | |
| --- | --- | --- | --- | --- | --- | --- | --- | --- | --- | --- |
|  |  | Mixed dementia | Unspecified dementia | AD | PDD | FTD | DLB | Other dementias | VaD | MCI |
| Baseline dementia diagnosis | Mixed dementia (8536) | 8,370 (98.0) | 30 (0.4) | 62 (0.7) | 2 (0) | 5 (0.1) | 20 (0.2) | 4 (0) | 33 (0.4) | 12 (0.1) |
|  | Unspecified dementia (10,607) | 216 (2) | 9,383 (88.5) | 535 (5.0) | 15 (0.1) | 43 (0.4) | 68 (0.6) | 40 (0.4) | 274 (2.6) | 33 (0.3) |
|  | AD (18,724) | 117 (0.6) | 67 (0.4) | 1,8400 (98.3) | 7 (0) | 14 (0.1) | 36 (0.2) | 14 (0.1) | 24 (0.1) | 45 (0.2) |
|  | PDD (772) | 1 (0.1) | 5 (0.7) | 3 (0.4) | 653 (97.2) | 1 (0.1) | 6 (0.9) | 0 (0) | 3 (0.4) | 0 (0) |
|  | FTD (828) | 4 (0.5) | 4 (0.5) | 11 (1.3) | 0 (0) | 797 (96.3) | 2 (0.2) | 5 (0.6) | 1 (0.1) | 4 (0.5) |
|  | DLB (1217) | 5 (0.4) | 11 (0.9) | 7 (0.6) | 2 (0.2) | 4 (0.3) | 1,179 (96.9) | 3 (0.2) | 4 (0.3) | 2 (0.2) |
|  | Other dementias (1142) | 15 (1.3) | 28 (2.5) | 36 (3.2) | 1 (0.1) | 7 (0.6) | 10 (0.9) | 1,011 (88.5) | 29 (2.5) | 5 (0.4) |
|  | VaD (6705) | 125 (1.9) | 34 (0.5) | 43 (0.6) | 3 (0) | 2 (0) | 10 (0.1) | 9 (0.1) | 6,459 (96.3) | 20 (0.3) |
| Green diagonal cells indicate n (%) of participants that retained their same diagnosis from baseline to first follow-up (median, 11 months).  Abbreviations: AD, Alzheimer’s dementia; PDD, Parkinson's Disease Dementia; FTD, Frontotemporal Dementia; LBD, Lewy Body Dementia; VaD, Vascular Dementia; MCI, Mild Cognitive Impairment. | | | | | | | | | | |


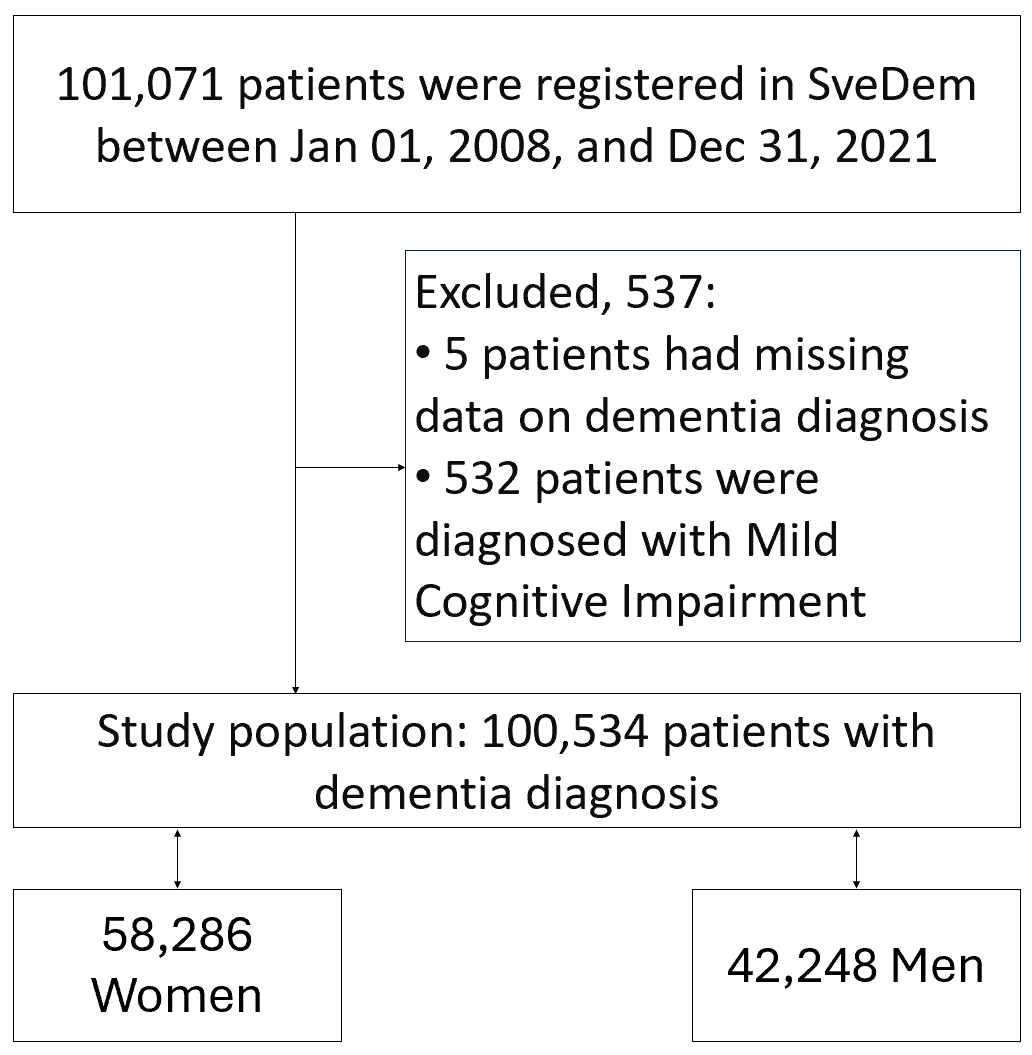


**Supplementary Figure 1.** Flowchart of the baseline study population. SveDem, Swedish Quality Registry for Cognitive/Dementia Disorders.

**
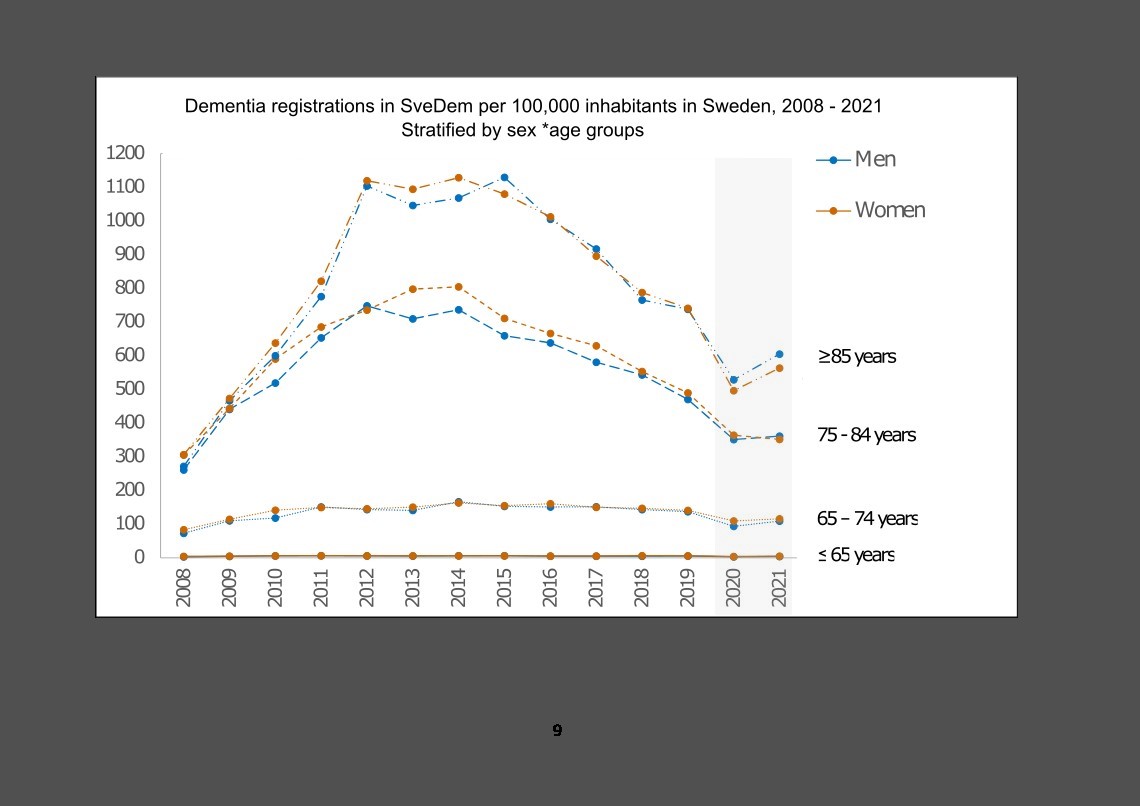
**

**Supplementary Figure 2.** Dementia registrations in the Swedish Quality Registry for Cognitive/Dementia Disorders (SveDem) per 100,000 Swedish inhabitants during 2008–2021 for men and women, stratified by age group. All participants with dementia registered in the SveDem were included. The gray box highlights the COVID-19 pandemic period in Sweden.

**
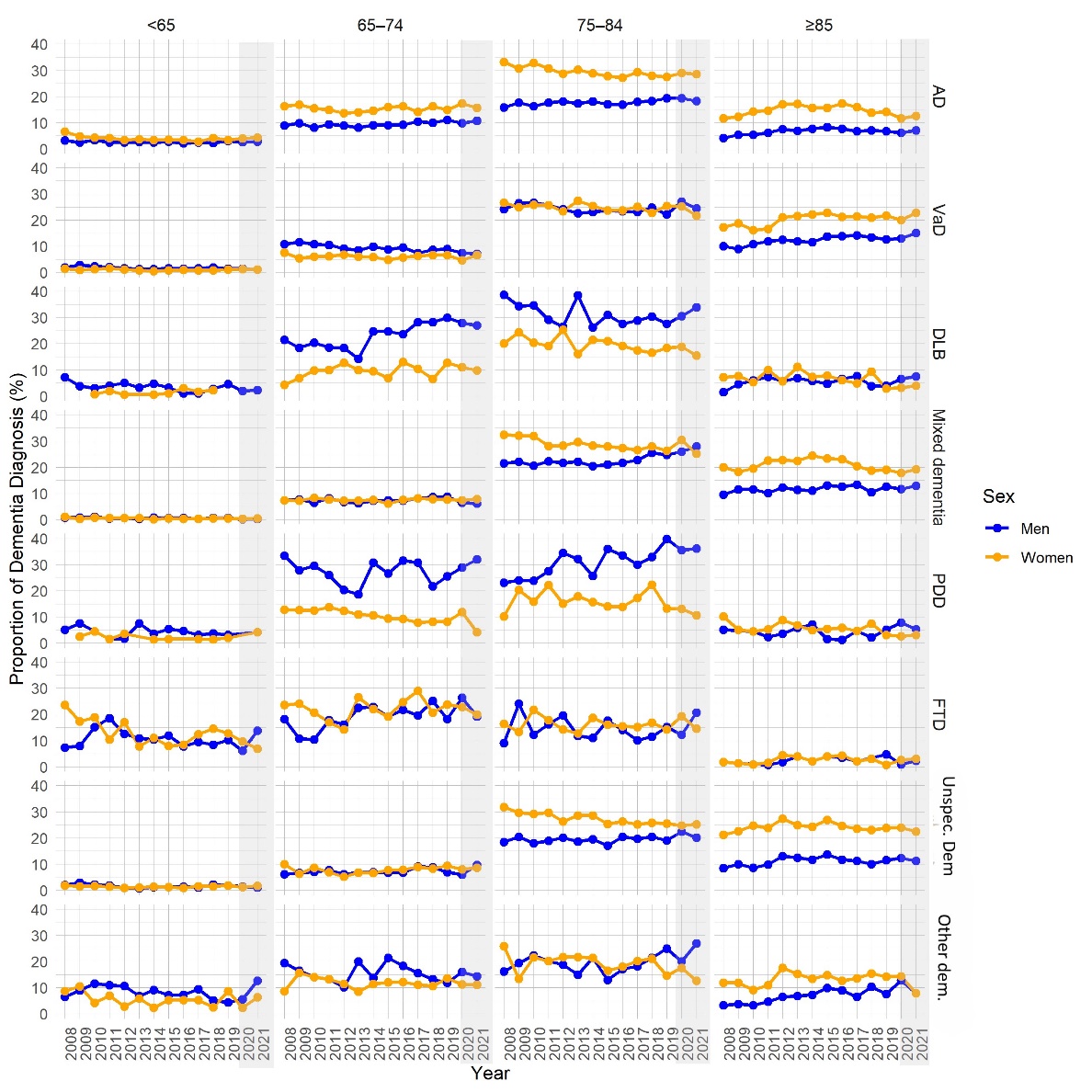
**

**Supplementary Figure 3.** Proportion of Dementia Diagnoses in the Swedish Quality Registry for Cognitive/Dementia Disorders by type, sex, and year (2008–2021), stratified by age group. Abbreviations: AD, Alzheimer’s Disease; VaD, Vascular Dementia; DLB, Dementia with Lewy Bodies; PDD, Parkinson’s Disease Dementia; FTD, Frontotemporal Dementia, Dem, Dementia; Unspec. Dem., unspecified dementia. The grey box indicates the COVID-19 period in Sweden.

**
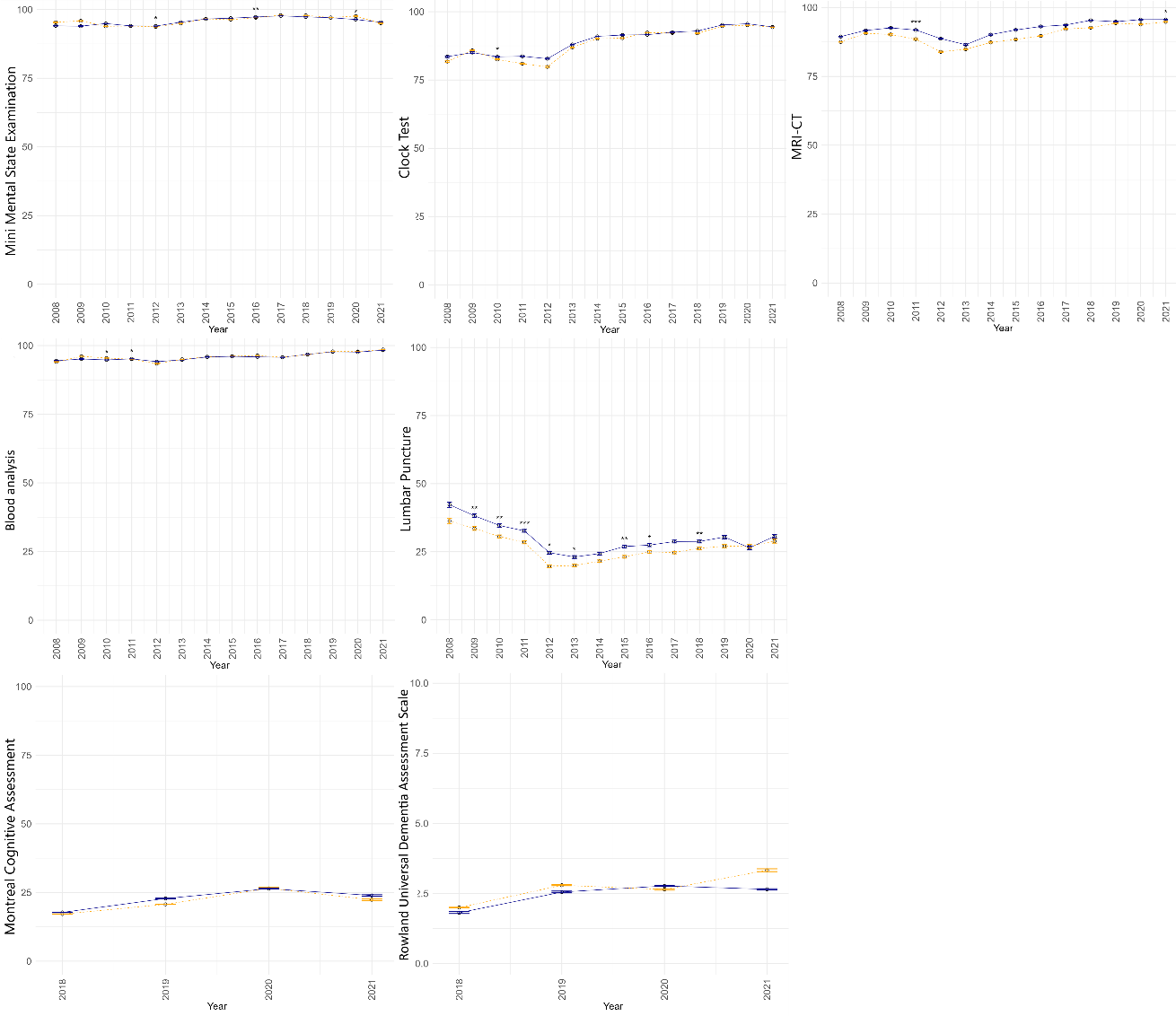
**

**Supplementary Figure 4.** Overall estimated proportions of diagnostic workups with associated 95% confidence intervals (2008–2021) on the Y-axis, by sex (orange line, women; blue line, men).
Abbreviations: MRI-CT, Magnetic Resonance Imaging-Computed Tomography. *p ≤ 0.05, **p ≤ 0.01, **p ≤ 0.001. Montreal cognitive assessment and Rowland Universal Dementia Assessment Scale scores were calculated for 2018–2021, owing to limited data availability for previous years. 2020–2021: the COVID-19 pandemic years in Sweden.


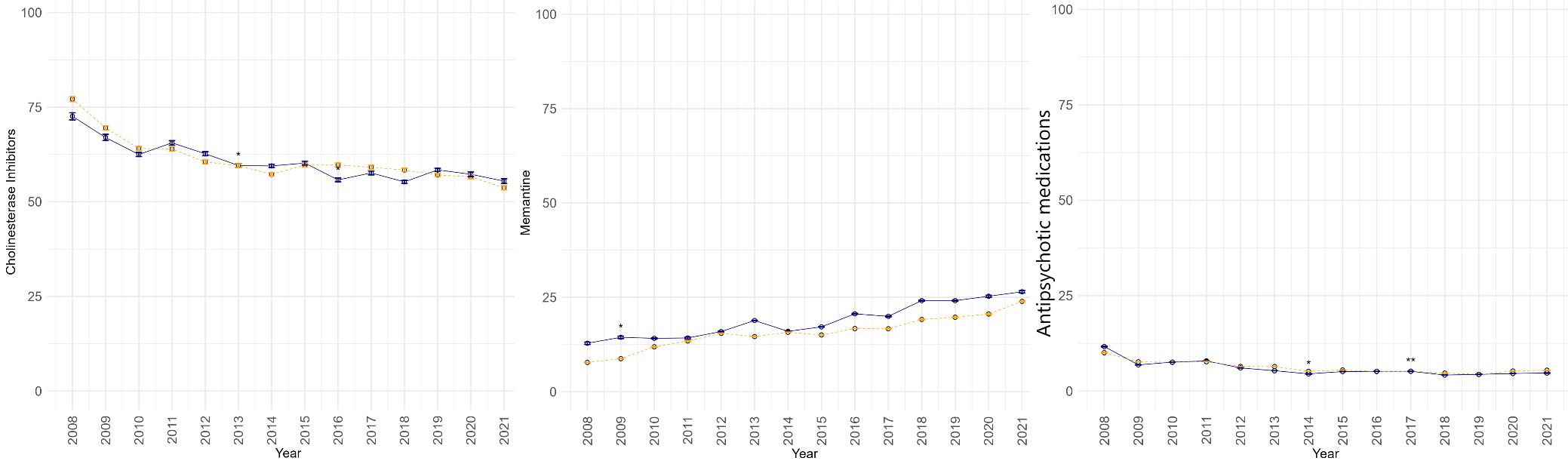


**Supplementary Figure 5.** Overall estimated proportions of medications registered in SveDem, with associated 95% confidence intervals (2008–2021) on the Y-axis, by sex (orange line, women; blue line, men).
*p ≤ 0.05, **p ≤ 0.01.
Memantine and Cholesterol-lowering: calculated on a subgroup of patients with Alzheimer's dementia and Mixed dementia. 2020–2021: the COVID-19 pandemic years in Sweden.


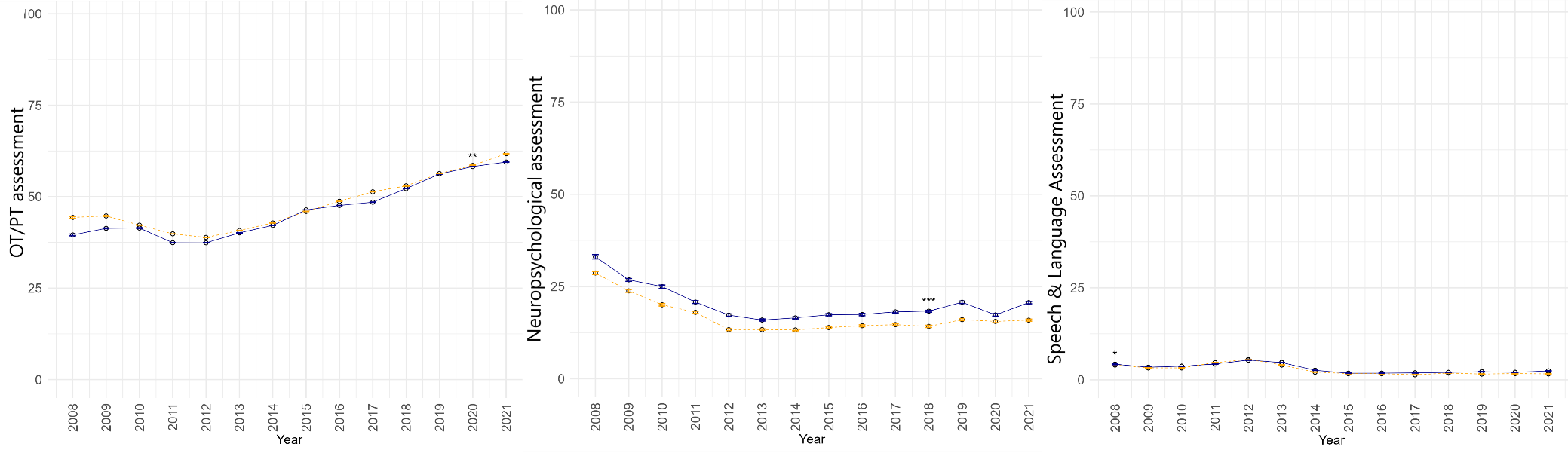


**Supplementary Figure 6.** Overall estimated proportions of assessments performed by healthcare professionals in SveDem, with associated 95% confidence intervals (2008–2021) on the Y-axis, by sex (orange line, women; blue line, men). *p ≤ 0.05, **p ≤ 0.01, **p ≤ 0.001. 2020–2021: COVID-19 pandemic years in Sweden.OT, Occupational therapist; PT, Physiotherapist.


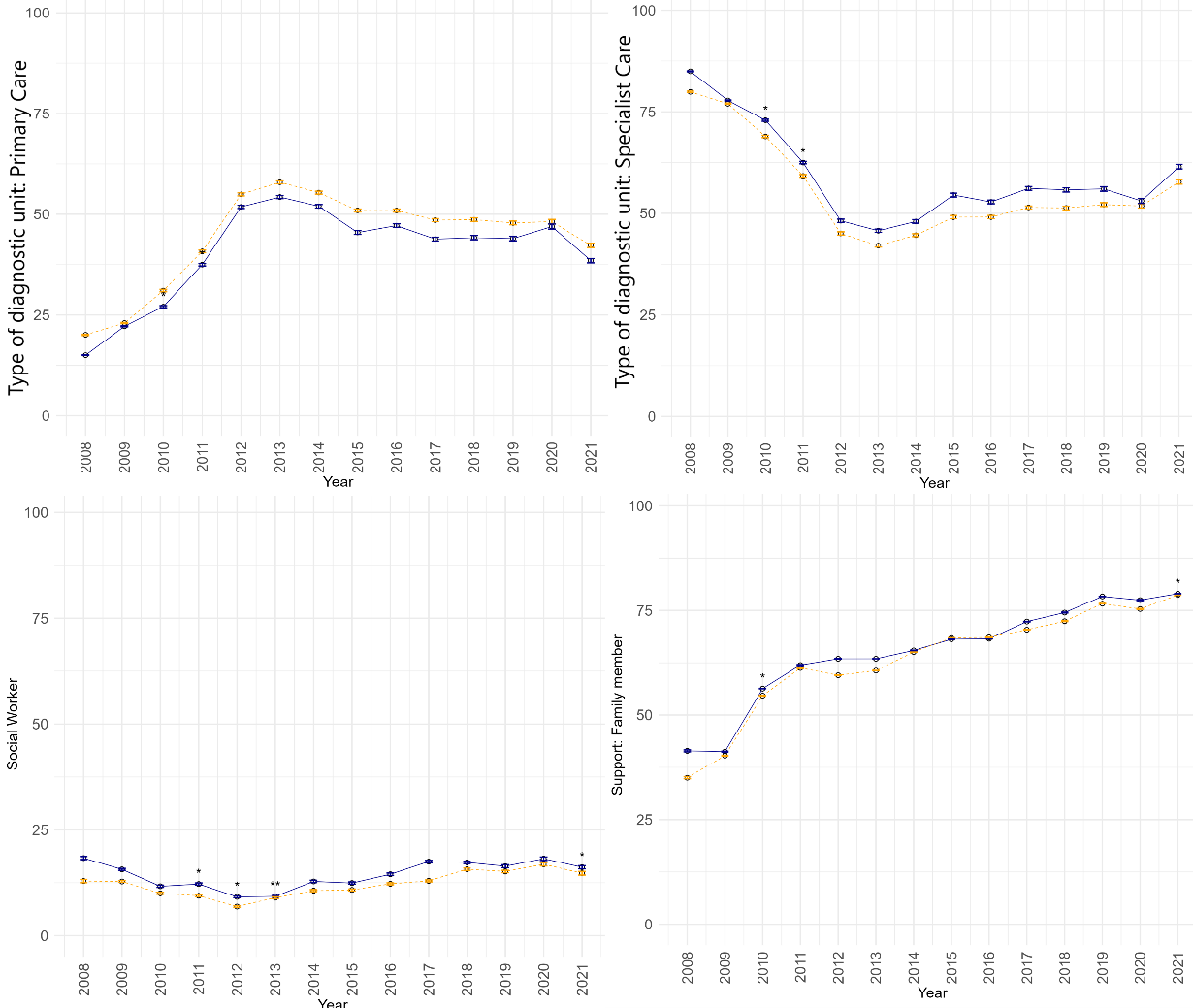


**Supplementary Figure 7.** Overall estimated proportions of support, with associated 95% confidence intervals (2008–2021) on the Y-axis, by sex (orange line, women; Blue line, men). *p ≤ 0.05, **p ≤ 0.01.
